# Urinary Red Blood Cell Distribution for Detection of Glomerular Hematuria: A Biopsy-Based Comparison with Direct Microscopy

**DOI:** 10.3390/diagnostics16162491

**Published:** 2026-08-07

**Authors:** Nuttasith Larpparisuth, Paweena Kanogpotjananont, Kanit Reesukumal, Methawoot Khemmongkol, Gahwin Ruchikajorndech, Chanchira Janwijit, Peenida Skulratanasak

**Affiliations:** 1 Division of Nephrology, Department of Medicine, Faculty of Medicine Siriraj Hospital, Mahidol University, 2 Prannok Road, Bangkok Noi, Bangkok 10700, Thailand; kub-p-om@hotmail.com (P.K.); m.khemmongkon@gmail.com (M.K.); gahwinr@gmail.com (G.R.); chanchira122@gmail.com (C.J.); p.skulratanasak@gmail.com (P.S.); 2 Department of Clinical Pathology, Faculty of Medicine Siriraj Hospital, Mahidol University, 2 Prannok Road, Bangkok Noi, Bangkok 10700, Thailand; kanit.ree@mahidol.ac.th

**Keywords:** diagnostic tests, routine, glomerulonephritis, hematuria, microscopy, red blood cells, urinalysis

## Abstract

**Background:** Differentiating glomerular from non-glomerular hematuria remains challenging. Automated urine analyzers with red blood cell (RBC) discrimination algorithms may provide an adjunct to conventional urine microscopy. We evaluated the diagnostic performance of the UF-5000 analyzer for detecting glomerular hematuria (GH). **Methods:** In this cross-sectional diagnostic study, patients with hematuria were classified into a GH group with biopsy-proven glomerular disease and a non-glomerular hematuria (NGH) group with non-glomerular causes confirmed by clinical, radiological, and/or pathological evaluation. Urine specimens were analyzed by a technician, a nephrology fellow, and the UF-5000 analyzer. Diagnostic performance was assessed using receiver operating characteristic (ROC) curve analysis and multivariable logistic regression. **Results:** A total of 201 patients were included, comprising 101 in the GH group and 100 in the NGH group. Urinary RBC distribution (URD) from UF-5000 analyzer was significantly higher in the GH group (66.3 ± 23.6 vs. 12.6 ± 19.8%; *p* < 0.0001). URD demonstrated the highest diagnostic performance among evaluated UF-5000 parameters, with an area under the ROC curve (AUC) of 0.946 (95% CI 0.914–0.979). The optimal Youden-derived URD cutoff ≥ 25% yielded 91.1% sensitivity and 89.0% specificity for identifying GH. Compared with microscopy, URD showed higher sensitivity than technician-performed microscopy (91.1% vs. 60.4%; *p* < 0.001) and similar performance to fellow-performed microscopy (91.1% vs. 83.2%; *p* = 0.237). Performance remained consistent across RBC count strata and major glomerular disease subtypes. **Conclusions:** URD was strongly associated with GH and demonstrated good discriminatory performance in this biopsy-based cohort. Automated urinary RBC analysis may complement conventional urine microscopy in hematuria evaluation.

## 1. Introduction

Glomerular diseases are a leading cause of chronic kidney disease and progression to end-stage kidney disease [1,2]. Microscopic hematuria is often one of the earliest clinical manifestations and remains a key clue for differentiating glomerular from non-glomerular pathology. In routine practice, this distinction relies on urine sediment examination using phase-contrast microscopy. Dysmorphic red blood cells (RBCs), particularly acanthocytes, are strongly associated with a glomerular source of bleeding [3,4]. Conventionally, glomerular hematuria is defined by acanthocytes exceeding 5% of urinary erythrocytes [5,6]. Despite its diagnostic value, manual microscopy is time-consuming, requires adequate urinary RBC counts, and is dependent on examiner expertise, resulting in inter-observer variability and potential delays in diagnosis [7].

Automated urine particle analyzers based on flow cytometry have been introduced as an alternative approach for urinary sediment analysis [7,8,9,10]. Earlier-generation analyzers demonstrated variable performance in identifying dysmorphic RBCs, and manual microscopy remained the reference method [11,12,13,14]. The third-generation Sysmex UF-5000 analyzer incorporates optical and fluorescence flow cytometry and provides several quantitative RBC-related parameters, including urinary RBC distribution (URD), percentage of small RBCs, percentage of lysed RBCs, RBC forward-scattered light intensity at the 70th percentile (RBC-P70 Fsc) and RBC surface-channel forward-scattered light distribution width (RBC-SF-Fsc-DW). These analyzer-derived parameters can be used to calculate urinary RBC distribution (URD), which may reflect morphological differences between glomerular hematuria (GH) and non-glomerular hematuria (NGH) [15].

Previous studies have reported associations between UF-5000-derived parameters, particularly URD, and the presence of GH [15,16,17]. However, most studies lacked biopsy-confirmed glomerular disease, focused on selected disease entities such as IgA nephropathy, or did not include direct comparisons with conventional urine microscopy performed by both laboratory personnel and clinicians [15,16,17]. Therefore, this study aimed to evaluate the diagnostic performance of UF-5000 urinary RBC parameters for detecting GH in patients with biopsy-confirmed glomerular disease, and to compare their performance with direct microscopic examination performed by experienced medical technologists and nephrology fellows.

## 2. Materials and Methods

### 2.1. Study Design and Population

This investigator-initiated, single-center, cross-sectional diagnostic accuracy study was conducted at Siriraj Hospital, Bangkok, Thailand, between 1 August 2023 and 31 January 2024. The study protocol was approved by the Institutional Review Board of the Faculty of Medicine Siriraj Hospital, Mahidol University (COA Si 338/2023). Written informed consent was obtained from all participants prior to enrollment. Adult patients (≥18 years) with hematuria were screened for eligibility. Inclusion criteria were urinalysis demonstrating ≥ 20 red blood cells (RBCs)/µL by automated analysis or ≥5 RBCs/high-power field (HPF) on direct microscopic examination.

Participants were classified into two groups according to the reference diagnosis. The GH group was defined using kidney biopsy as the reference standard and consisted of patients with biopsy-proven glomerular disease. The NGH group included patients with hematuria attributable to non-glomerular causes, classified using a composite clinical reference standard based on clinical evaluation, urological assessment, imaging studies (ultrasonography, computed tomography, or magnetic resonance imaging), and/or urological histopathology when available. No patient classified as NGH underwent kidney biopsy, as kidney biopsy was not clinically indicated or ethically justified in patients with established non-glomerular causes of hematuria. The reference diagnosis was established using clinical, pathological, and radiological criteria, and UF-5000 results were not used for case classification. Patients with uncertain or overlapping diagnoses, including those in whom a glomerular source of hematuria could not be excluded, were excluded. Patients with active pyelonephritis, menstruation at the time of urine collection, bleeding from adjacent organs, or unwillingness to participate were also excluded.

Based on a projected AUC of 0.90 and a null hypothesis AUC of 0.75, approximately 90 patients per group would provide 80% power at a two-sided α level of 0.05. After accounting for an anticipated attrition rate of 10–15%, a target sample size of 100–110 patients per group was considered appropriate.

### 2.2. Urine Sample Analysis

Urine specimens from each participant were thoroughly mixed before aliquoting to minimize sampling variability and were subsequently analyzed independently by the following three methods: (1) automated urine analysis using the Sysmex UF-5000 analyzer (Sysmex, Kobe, Japan), (2) direct microscopic examination by a technician (C.J.), and (3) direct microscopic examination by a nephrology fellow (M.K.). All analyses were performed using aliquots obtained from the same freshly collected urine specimen. Automated analysis using the UF-5000 was performed first, followed by conventional microscopic examination of the remaining aliquots. Both the technologist and fellow were blinded to the clinical diagnosis and UF-5000 results.

The automated urine analyzer UF-5000 is a flow cytometry-based system incorporating optical and fluorescence signals. The analyzer provides several RBC-related parameters, including percentage of small RBCs, percentage of lysed RBCs, RBC-P70 Fsc, and RBC-SF-Fsc-DW. In this study, we evaluated these analyzer-derived variables together with URD, which was calculated from the percentages of small RBCs and lysed RBCs and reflects the relative distribution or heterogeneity of urinary RBC size. These parameters were assessed for their ability to differentiate GH from NGH.

Both UF-5000 analysis and microscopic examination were performed within 2 h of urine collection. Prior to study initiation, the technologist and nephrology fellow reached consensus on standardized criteria for RBC morphology assessment. GH by conventional microscopy was defined as the presence of dysmorphic RBCs with acanthocytes accounting for ≥5% of urinary RBCs.

Urinary protein concentration was measured using the pyrogallol red–molybdate method, urinary albumin was measured using an immunoturbidimetric assay, and urinary creatinine (mg/dL) was measured using an enzymatic method. The urinary albumin-to-creatinine ratio (UACR) was calculated as urinary albumin concentration divided by urinary creatinine concentration, and the urine protein-to-creatinine ratio (UPCR) was calculated as urinary protein concentration divided by urinary creatinine concentration. All urine chemistry analyses were performed using the Roche Cobas C702 analyzer (Roche Diagnostics, Indianapolis, IN, USA).

### 2.3. Statistical Analysis

Continuous variables are presented as mean ± standard deviation or median (interquartile range; IQR), as appropriate. Categorical variables are expressed as frequencies and percentages. Between-group comparisons were performed using the independent *t*-test or Mann–Whitney U test for continuous variables and the chi-square test for categorical variables.

The diagnostic performance of UF-5000 parameters for identifying glomerular hematuria was evaluated using receiver operating characteristic (ROC) curve analysis. Areas under the ROC curve (AUCs) with 95% confidence intervals (CIs) were calculated and compared. The optimal cutoff value for each UF-5000 parameter was determined using the Youden index. Sensitivity, specificity, positive likelihood ratio (LR+), and negative likelihood ratio (LR–) were calculated at the selected cutoff values. To assess the robustness of the study-derived URD cutoff, internal validation was performed using bootstrap resampling with 5000 iterations. Bootstrap-derived 95% confidence intervals were calculated for AUC, sensitivity, and specificity.

Univariate logistic regression analysis was initially performed to identify factors associated with GH. A multivariable logistic regression model was subsequently constructed to evaluate whether URD remained independently associated with GH after adjustment for clinically relevant covariates, including age, sex, diabetes mellitus, malignancy, serum creatinine (SCr) and UPCR. Multicollinearity among predictor variables was assessed using variance inflation factors (VIFs), with VIF values > 5 considered indicative of significant collinearity.

Agreement between technician-performed microscopy, fellow-performed microscopy, UF-5000 classification, and biopsy-confirmed diagnosis was evaluated using Cohen’s kappa (κ) statistic with corresponding 95% confidence intervals. All statistical analyses were conducted using SPSS Statistics version 29.0 (IBM Corp., Armonk, NY, USA) and MedCalc version 19.5.0 (MedCalc Software Ltd., Ostend, Belgium). A two-sided *p* value < 0.05 was considered statistically significant.

## 3. Results

A total of 201 patients were enrolled, including 101 patients in the GH group and 100 patients in the NGH group. Patients in the GH group were younger than those in the NGH group (mean age 47.2 ± 18.2 vs. 64.3 ± 16.6 years; *p* < 0.001), and female patients were more prevalent (61.4% vs. 28%; *p* < 0.001). Baseline SCr was comparable between the GH and NGH groups (2.6 ± 2.5 vs. 2.4 ± 2.4 mg/dL; *p* = 0.456). BUN was higher in the GH group than in the NGH group (40.9 ± 28.7 vs. 30.7 ± 25.7 mg/dL; *p* = 0.009). UPCR and UACR were significantly higher in GH group than in the NGH group (UPCR 3.8 ± 3.8 vs. 0.9 ± 1.2 g/gCr; UACR 2081 ± 2229 vs. 289 ± 452 mg/gCr; both *p* < 0.001). Serum albumin was significantly lower in the GH group than in the NGH group (2.9 ± 0.8 vs. 3.4 ± 0.8 g/dL; *p* < 0.001), whereas hemoglobin and mean corpuscular volume were comparable between the two groups (Table 1).

**Figure 1:**
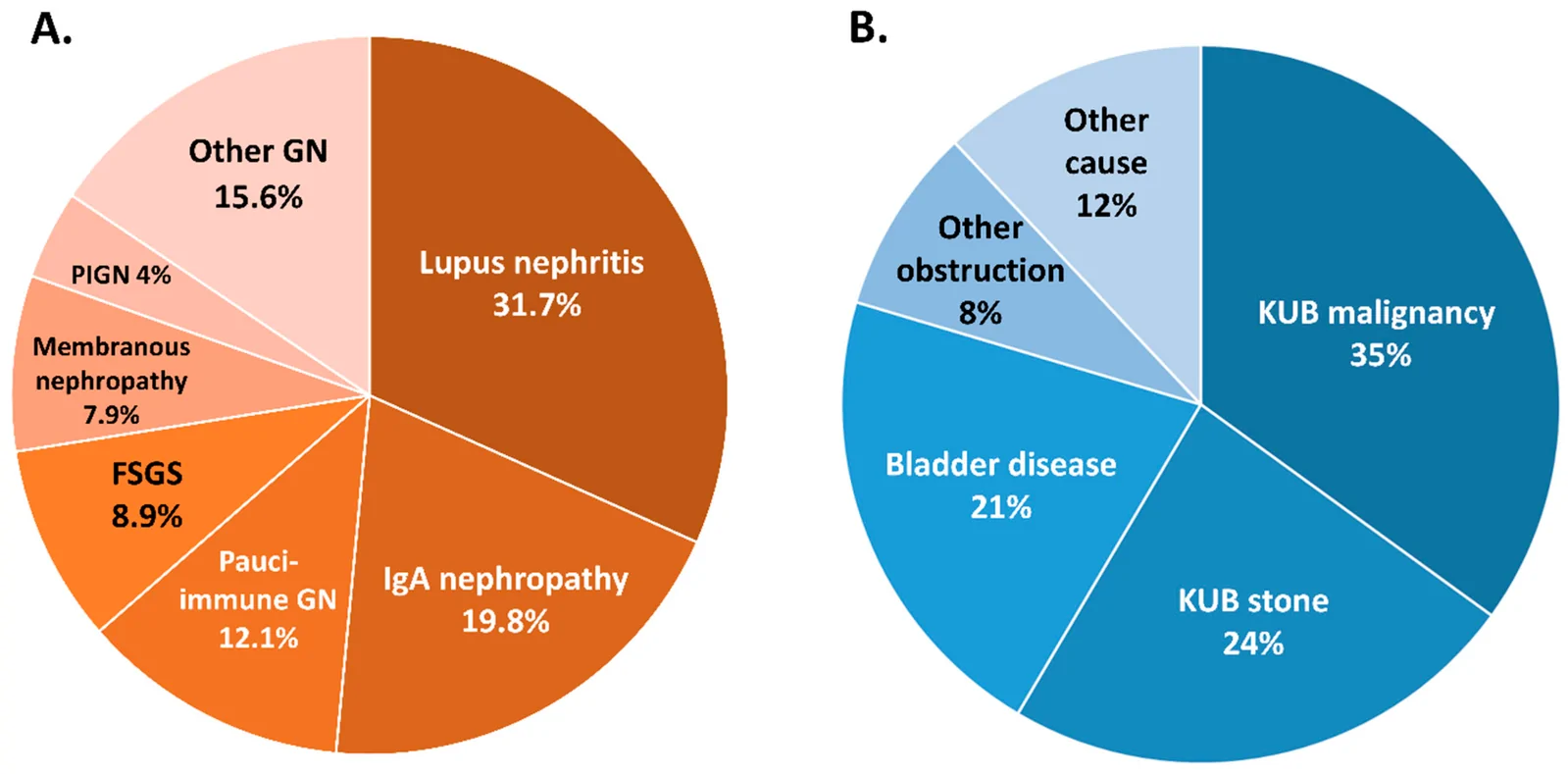
Distribution of underlying diagnoses among patients with glomerular hematuria (GH) and non-glomerular hematuria (NGH). (**A**) Distribution of biopsy-proven glomerular diseases in the GH group (*n* = 101), including lupus nephritis (*n* = 32), IgA nephropathy (*n* = 20), pauci-immune glomerulonephritis (*n* = 13), focal segmental glomerulosclerosis (*n* = 9), membranous nephropathy (*n* = 8), post-infectious glomerulonephritis (*n* = 4), and other glomerular diseases (*n* = 15). (**B**) Distribution of underlying etiologies in the NGH group (*n* = 100), including malignancies of the kidney or urinary tract (*n* = 35), urolithiasis (*n* = 24), non-malignant bladder diseases (*n* = 21), urinary tract obstruction requiring intervention (*n* = 8), and other urological conditions or post-procedural states (*n* = 12). GH, glomerular hematuria; NGH, non-glomerular hematuria.

In the GH group, common clinical presentations included new onset or worsening hypertension (55.4%), edema (59.8%), rapidly declining kidney function (21.8%), and gross hematuria (16.8%). Definite diagnoses in the glomerular group were lupus nephritis (31.7%), IgA nephropathy (19.8%), pauci-immune glomerulonephritis (12.1%), focal segmental glomerulosclerosis (8.9%), membranous nephropathy (7.9%), post-infectious glomerulonephritis (PIGN) (4.0%), anti–glomerular basement membrane disease (3.0%), and other diagnoses (12.6%) (Figure 1A).

In the NGH group, causes of hematuria included malignancy of the kidney or urinary tract (35%), urolithiasis (24%), non-malignant bladder disease (21%), urinary tract obstruction requiring intervention (8%), and other urological conditions or post-procedural states (12%) (Figure 1B). Among NGH patients with elevated SCr, renal dysfunction was largely attributable to post-renal acute kidney injury or chronic obstructive uropathy related to underlying urological disease.

### 3.1. UF-5000 Urinary RBC Parameters

UF-5000-derived urinary RBC parameters differed significantly between the GH and NGH groups. The mean URD was markedly higher in the GH group than in the NGH group (66.3 ± 23.6% vs. 12.6 ± 19.8%, *p* < 0.0001). Parameters reflecting RBC heterogeneity, including percentage of small RBCs, percentage of lysed RBCs, and RBC-SF-Fsc-DW, were also significantly higher in GH group, whereas RBC-P70 Fsc values were significantly lower (all *p* < 0.0001) (Table 2).

**Figure 2:**
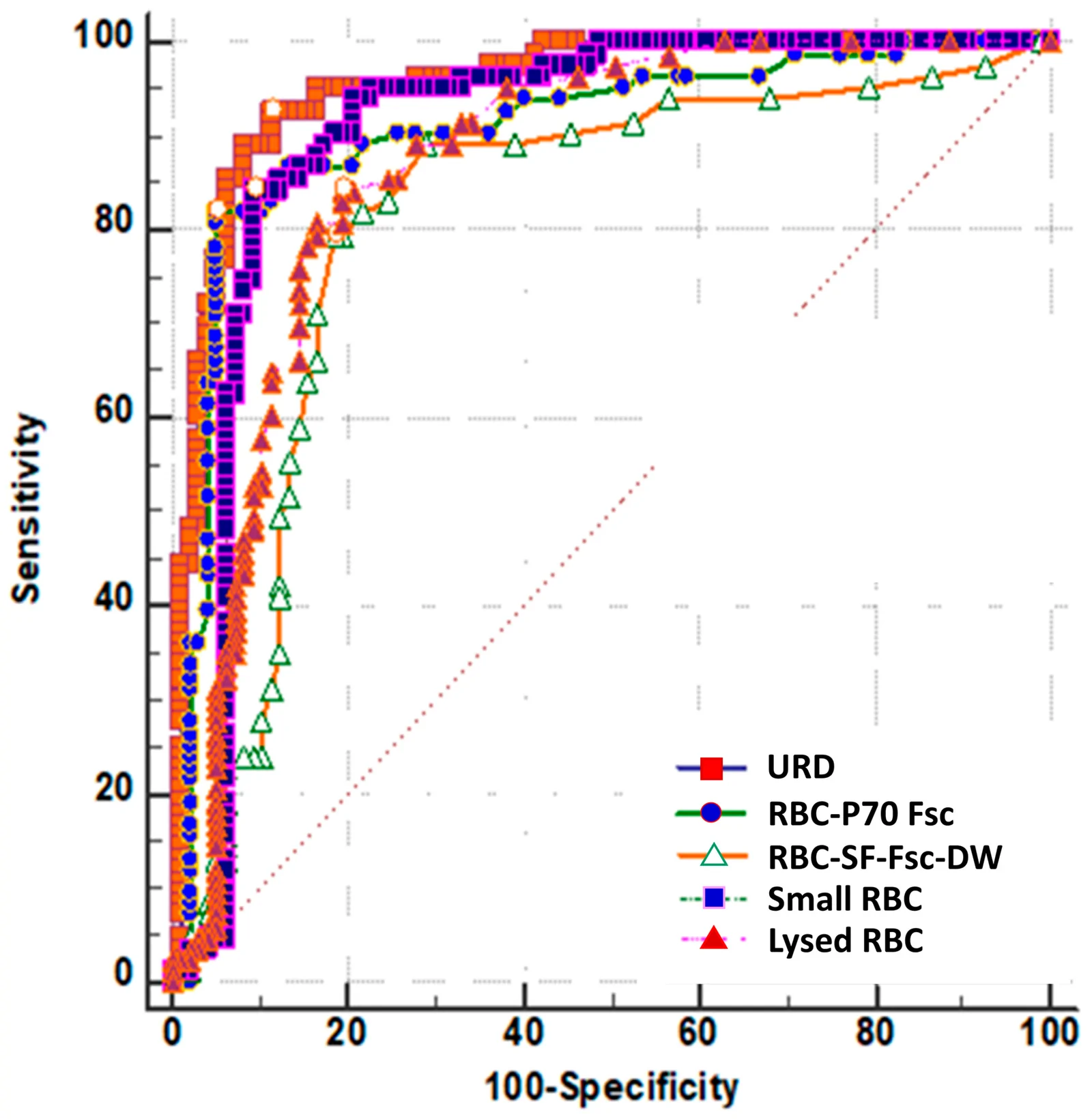
Receiver operating characteristic (ROC) curves of UF-5000 urinary red blood cell parameters for differentiating glomerular from non-glomerular hematuria. Among the evaluated UF-5000 parameters, URD demonstrated the highest diagnostic performance (AUC 0.946, 95% CI 0.914–0.979), followed by RBC-P70 Fsc (AUC 0.906, 95% CI 0.858–0.954), small RBC percentage (AUC 0.902, 95% CI 0.853–0.952), lysed RBC percentage (AUC 0.868, 95% CI 0.816–0.921), and RBC-SF-Fsc-DW (AUC 0.811, 95% CI 0.743–0.879). AUC, area under the curve; ROC, receiver operating characteristic; URD, urinary red blood cell distribution; RBC-P70 Fsc, red blood cell forward-scattered light intensity at the 70th percentile; RBC-SF-Fsc-DW, RBC surface-channel forward-scattered light distribution width; small RBC, percentage of small red blood cells; lysed RBC, percentage of lysed red blood cells.

Receiver operating characteristic (ROC) analysis demonstrated that URD provided the highest diagnostic accuracy for detecting GH (AUC 0.946). The optimal cutoff determined by the Youden index was URD ≥ 25%. Internal validation using 5000 bootstrap resamples supported the stability of this study-derived cutoff, with a bootstrap-corrected sensitivity of 91.1% (95% CI 85.0–96.2%) and specificity of 89.0% (95% CI 82.3–94.7%). At this threshold, the positive likelihood ratio (LR+) was 8.28 and the negative likelihood ratio (LR−) was 0.10. The corresponding bootstrap 95% confidence interval for AUC was 0.910–0.975 (Supplementary Table S1). Other UF-5000 parameters showed lower discriminatory performance, including RBC-P70 Fsc < 125 ch (AUC 0.906), %small RBC ≥ 35% (AUC 0.902), %lysed RBC ≥ 2% (AUC 0.868), and RBC-SF-Fsc-DW ≥ 40 ch (AUC 0.811) (Figure 2). Combining multiple UF-5000 parameters did not improve diagnostic performance beyond URD alone. The associations between individual UF-5000 urinary RBC parameters and glomerular hematuria are summarized in Supplementary Table S2.

To evaluate whether the diagnostic performance of URD was independent of baseline demographic differences between study groups, a multivariable logistic regression model adjusted for age, sex, diabetes mellitus, malignancy, SCr and UPCR was constructed. URD remained independently associated with glomerular hematuria after adjustment (adjusted OR 1.08 per 1% increase, 95% CI 1.06–1.11; *p* < 0.001) (Table 3). Variance inflation factor analysis demonstrated no significant multicollinearity among model covariates (all VIFs < 5.0) (Supplementary Table S3). Similar findings were observed when URD was analyzed as a dichotomous variable using the study-derived cutoff of ≥25% (adjusted OR 58.4, 95% CI 18.7–182.5; Supplementary Table S4). In an additional sensitivity analysis including BUN in the multivariable model, URD remained independently associated with GH (adjusted OR 1.07 per 1% increase, 95% CI 1.04–1.09; *p* < 0.001), whereas BUN was not independently associated with GH (adjusted OR 1.00, 95% CI 0.97–1.03; *p* = 0.888) (Supplementary Table S5).

The diagnostic performance of URD ≥ 25% was consistent across major glomerular disease subtypes, including IgA nephropathy, lupus nephritis, pauci-immune glomerulonephritis, focal segmental glomerulosclerosis, and membranous nephropathy, with sensitivities ranging from 85.0% to 100.0% without statistically significant differences across disease subtypes (Table 4). Diagnostic performance of URD ≥ 25% remained consistent across urinary RBC count strata, with AUC values ranging from 0.933 to 1.000. No apparent reduction in sensitivity or specificity was observed at lower RBC counts (Supplementary Table S6).

### 3.2. Comparison with Conventional Urine Microscopy

**Figure 3:**
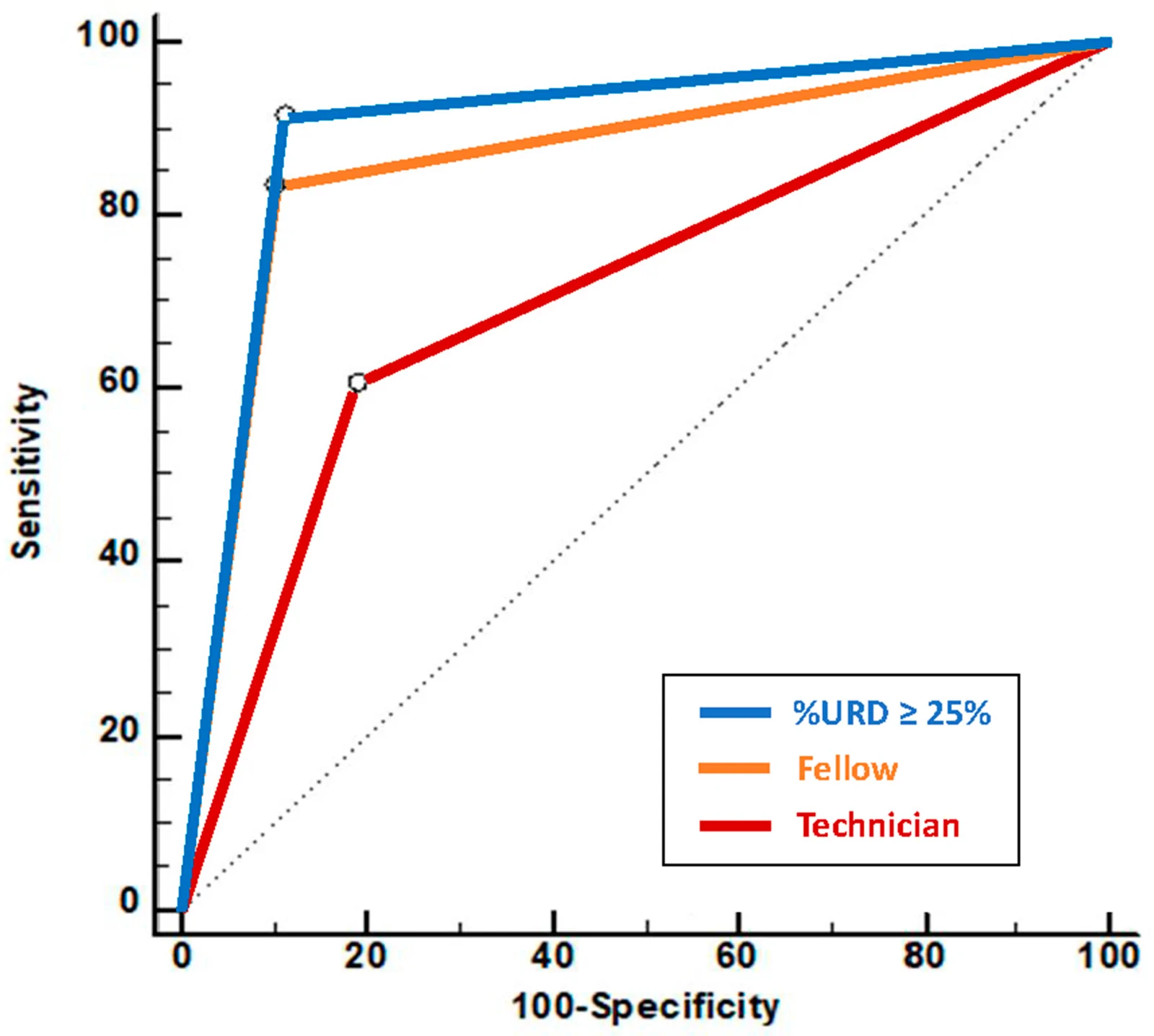
Comparison of diagnostic performance between urinary red blood cell distribution (URD) ≥ 25%, technician-performed microscopy, and fellow-performed microscopy for the detection of glomerular hematuria. URD ≥ 25% achieved a sensitivity of 91.1% and specificity of 89.0%, compared with 83.2% and 90.0% for fellow-performed microscopy and 60.4% and 81.0% for technician-performed microscopy. The diagonal line represents the performance of a non-discriminatory test. URD, urinary red blood cell distribution.

Using acanthocyte ≥ 5% as the morphologic criterion for glomerular hematuria, technician-performed microscopy yielded a sensitivity of 60.4% and specificity of 81.0%, whereas fellow-performed microscopy achieved a sensitivity of 83.2% and specificity of 90.0% (Table 5A). The diagnostic accuracy of URD ≥ 25% was superior to that of the technologist (*p* < 0.0001) and comparable to that of the nephrology fellow (*p* = 0.24), with the highest AUC observed for UF-5000–derived URD (Table 5B, Figure 3). Distribution of acanthocyte grades is shown in Supplementary Table S7. URD ≥ 25% demonstrated significant correlations with acanthocyte burden assessed by both technician-performed microscopy (Spearman’s ρ = 0.337, *p* < 0.001) and fellow-performed microscopy (ρ = 0.598, *p* < 0.001).

Inter-observer agreement between technician- and fellow-performed microscopy was κ = 0.637 (0.532–0.741). Agreement with biopsy-confirmed glomerular hematuria was κ = 0.414 (0.287–0.533) for technician-performed microscopy and κ = 0.731 (0.631–0.821) for fellow-performed microscopy. URD ≥ 25% demonstrated κ = 0.801 (0.720–0.871) with biopsy-confirmed diagnosis and κ = 0.652 (0.544–0.752) with fellow-performed microscopy (Table 5C).

## 4. Discussion

In this biopsy-based diagnostic study, URD demonstrated the highest diagnostic performance among the evaluated UF-5000 parameters for differentiating GH from NGH. URD achieved an AUC of 0.946 and remained independently associated with GH after adjustment for demographic and clinical factors. The optimal Youden-derived cutoff of URD ≥ 25% in this study provided high sensitivity and specificity and maintained consistent diagnostic performance across different urinary RBC count strata and major glomerular disease subtypes.

Patients with GH also exhibited significantly higher urinary protein excretion, consistent with glomerular filtration barrier injury resulting in both increased protein permeability and alterations in urinary RBC morphology [18,19]. Notably, URD remained independently associated with GH after adjustment for proteinuria and other clinical covariates, suggesting that its diagnostic value was not solely explained by the presence of increased urinary protein excretion. Nevertheless, proteinuria remains clinically relevant when evaluating hematuria and may provide complementary information when interpreted alongside urinary RBC morphology. Because proteinuria may also occur in non-glomerular conditions, particularly urological malignancies and obstructive uropathy, urinary protein indices should not be considered specific markers of glomerular bleeding and are best interpreted in conjunction with RBC morphology findings [20,21].

Among the evaluated UF-5000 parameters, URD demonstrated the highest discriminatory performance for differentiating GH from NGH. Diagnostic accuracy remained consistently high across different urinary RBC count strata, suggesting that URD retained discriminatory ability even in samples with relatively low erythrocyte concentrations. However, the number of patients in the lowest RBC-count stratum was limited and these findings should be interpreted with caution. In addition, URD correlated closely with acanthocyte burden assessed by urine microscopy, supporting its biological relevance as a quantitative marker of urinary RBC dysmorphism [10,15,16].

From a practical perspective, the use of a single URD threshold may facilitate interpretation of automated urine analysis in routine clinical practice, where clinicians often need to rapidly distinguish GH from NGH. The study-derived cutoff of URD ≥ 25% provided favorable sensitivity and specificity and demonstrated stable diagnostic performance in bootstrap internal validation, supporting the internal robustness of the selected threshold within our study population.

The optimal URD cutoff identified in the present study was lower than that reported by Cho et al. and Lv et al., who proposed thresholds of approximately 35–40% [15,16]. Several factors may explain this difference. Previous studies were largely based on clinical diagnoses and, in some cases, focused on selected disease entities such as IgA nephropathy [16]. In contrast, all patients in the present study had biopsy-confirmed glomerular disease and represented a broader spectrum of glomerular disorders. Differences in disease spectrum, patient characteristics, urinary RBC burden, and reference standards may therefore influence the distribution of urinary RBC morphology and the optimal URD threshold.

This broader disease representation may be particularly relevant because the underlying diagnosis is usually unknown when urine analysis is performed in routine clinical practice. Therefore, a cutoff derived from a heterogeneous biopsy-confirmed cohort may better reflect the real-world setting in which urinary RBC morphology is used to evaluate hematuria. Nevertheless, external validation in independent cohorts remains necessary before widespread implementation of a specific URD threshold.

Compared with conventional microscopy, URD demonstrated higher sensitivity than technician-performed microscopy and similar overall performance to fellow-performed microscopy. In addition, URD showed strong agreement with the reference diagnosis (κ = 0.801), whereas fellow-performed microscopy showed substantial agreement with the reference diagnosis (κ = 0.731). Agreement between URD and fellow-performed microscopy was also substantial (κ = 0.652), and agreement between technician- and fellow-performed microscopy was lower (κ = 0.637), highlighting the inherent variability of urinary sediment interpretation. These findings suggest that automated urinary RBC analysis may provide more standardized assessment of RBC morphology across different observers [6,7,17].

This potential advantage may be particularly relevant in routine clinical practice, where access to experienced urine microscopists is variable and laboratory workload is often high. In such settings, URD may provide useful adjunctive information to support the evaluation of hematuria. However, these findings should not be interpreted as evidence that automated analysis consistently outperforms expert urine microscopy in all settings. The diagnostic accuracy of urinary sediment examination remains highly dependent on operator training and experience, and experienced nephrologists or laboratory personnel may achieve diagnostic performance comparable to, or potentially exceeding, that observed with automated analysis. Therefore, automated urinary RBC analysis should be viewed as a complementary tool rather than a replacement for expert microscopic examination.

Several limitations should be acknowledged. First, the GH group was defined using kidney biopsy as the reference standard, whereas the NGH group was classified using a composite clinical reference standard based on clinical evaluation, urological assessment, imaging studies, and/or urological histopathology when available. No patient classified as NGH underwent kidney biopsy, as kidney biopsy was not clinically indicated or ethically justified in patients with established non-glomerular causes of hematuria. Therefore, differential verification bias may have been introduced, and subclinical glomerular disease within the NGH group cannot be completely excluded. Second, patients with uncertain or overlapping etiologies were excluded to establish clearly defined diagnostic groups. Although this approach strengthened internal validity, it may have resulted in a study population that does not fully reflect the complexity of real-world clinical practice, where mixed or indeterminate causes of hematuria are common. As a result, the diagnostic performance observed in this study may represent an estimate under relatively well-characterized clinical conditions. Third, this was a single-center cross-sectional diagnostic accuracy study, and the disease-subtype analyses should be considered exploratory because of the limited number of patients within each glomerular disease subtype. Although bootstrap resampling supported the internal robustness of the study-derived URD cutoff, this approach does not replace external validation. Therefore, validation in larger multicenter cohorts of consecutive hematuria patients is required before widespread clinical implementation.

Future studies should include patients with mixed or indeterminate etiologies, evaluate discordant or misclassified cases, and determine how URD may be incorporated into diagnostic algorithms together with proteinuria, kidney function, clinical context, and expert urine microscopy. Finally, although the study received research funding from the manufacturer of the UF-5000 analyzer, data collection, statistical analysis, interpretation of results, and manuscript preparation were performed independently by the investigators.

Despite these limitations, several aspects of the study strengthen the interpretation of our findings. First, all patients in the glomerular hematuria group had biopsy-confirmed glomerular disease, providing a well-defined reference diagnosis. Second, the study included a broad spectrum of glomerular disorders rather than a single disease entity [15,16,17], allowing evaluation of URD across clinically diverse presentations. Finally, the incorporation of direct comparisons with both technician- and fellow-performed urine microscopy, together with assessment across different urinary RBC count strata, provides additional context regarding the potential clinical utility of automated urinary RBC analysis.

## 5. Conclusions

URD demonstrated good diagnostic performance for differentiating glomerular from non-glomerular hematuria in a cohort that included biopsy-confirmed glomerular disease. Automated urinary RBC analysis may provide useful adjunctive information in the evaluation of hematuria, particularly in settings where expertise in urine microscopy is limited. Further external validation in larger multicenter cohorts of consecutive hematuria patients is needed to confirm the study-derived URD cutoff and determine its applicability in broader clinical practice.

## Figures and Tables

**Table 1:** Baseline demographic characteristics, underlying diseases, and laboratory profiles of all patients, stratified by GH and NGH groups.

Parameter	All Patients (*n* = 201)	Glomerular Hematuria (*n* = 101)	Non-Glomerular Hematuria (*n* = 100)	*p* Value
Age (years)	55.7 ± 19.4	47.2 ± 18.2	64.3 ± 16.6	<0.001
Female sex (%)	44.8	61.4	28	<0.001
Height (cm)	163.2 ± 9.2	162 ± 9.6	164.4 ± 8.6	0.06
Weight (kg)	61.9 ± 15.7	62.2 ± 16.4	61.6 ± 15.1	0.779
Underlying disease				
- Diabetes mellitus (%)	18.9	10.9	27	0.004
- Hypertension (%)	52.7	47.5	58	0.137
- Malignancy (%)	9.5	2	17	<0.001
- Cardiovascular disease (%)	10.4	4	17	0.003
**Laboratory profiles**				
BUN (mg/dL)	35.9 ± 27.7	40.9 ± 28.7	30.7 ± 25.7	0.009
Serum creatinine (mg/dL)	2.5 ± 2.5	2.6 ± 2.5	2.4 ± 2.4	0.456
UPCR (g/gCr)	2.1 ± 2.6	3.8 ± 3.8	0.9 ± 1.2	<0.001
UACR (mg/gCr)	718 ± 1324	2081 ± 2229	289 ± 452	<0.001
Urine RBC (cell/μL)	2246 ± 6539	515 ± 968	3993 ± 8905	<0.001
Hemoglobin (g/dL)	10.3 ± 2.1	10.2 ± 2.1	10.4 ± 2.2	0.476
MCV (fL)	83.8 ± 7.6	83.4 ± 8	84.2 ± 7.2	0.453
Serum albumin (g/dL)	3.2 ± 0.8	2.9 ± 0.8	3.4 ± 0.8	<0.001

Abbreviations: BUN, blood urea nitrogen; UPCR, urine protein creatinine ratio; UACR, urine albumin creatinine ratio; RBC, red blood cell; MCV, mean corpuscular volume.

**Table 2:** Distribution of UF-5000-derived urinary red blood cell parameters in patients with glomerular hematuria (GH) and non-glomerular hematuria (NGH), and their diagnostic performance for discrimination between the two groups.

UF-5000 Parameters	Glomerular Hematuria (*n* = 101)	Non-Glomerular Hematuria (*n* = 100)	*p* Value	AUC	95% CI
URD (%)	66.3 ± 23.6	12.6 ± 19.8	<0.001	0.946	0.914–0.979
RBC-P70 Fsc (ch)	96.3 ± 30.9	147.4 ± 23.9	<0.001	0.906	0.858–0.954
RBC-SF-Fsc-DW (ch)	58 ± 27.9	35 ± 24.6	<0.001	0.811	0.743–0.879
Small_RBC (%)	58.3 ± 22	11.6 ± 19.2	<0.001	0.902	0.853–0.952
Lysed_RBC (%)	9.4 ± 11.8	2.6 ± 6.6	<0.001	0.868	0.816–0.921

Values are presented as mean ± standard deviation. Diagnostic performance was assessed using receiver operating characteristic analysis, with area under the curve (AUC) and 95% confidence interval (CI) shown. UF-5000 parameters include urinary red blood cell distribution (URD), RBC-P70 forward-scattered light (RBC-P70 Fsc), RBC surface-channel forward-scattered light distribution width (RBC-SF-Fsc-DW), percentage of small red blood cells (Small_RBC), and percentage of lysed red blood cells (Lysed_RBC).

**Table 3:** Multivariable logistic regression analysis adjusted for demographic and clinical confounders.

Variable	Adjusted OR	95% CI	*p*
URD (%)	1.08	1.06–1.11	<0.001
Age	0.98	0.94–1.02	0.340
Female sex	0.67	0.15–3.01	0.597
Diabetes mellitus	0.27	0.05–1.33	0.107
Malignancy	0.08	0.007–0.84	0.036
Serum creatinine	1.12	0.88–1.44	0.356
Urine protein	2.27	1.41–3.64	<0.001

All variance inflation factors were <5.0, indicating no significant multicollinearity. Abbreviations: URD, urinary red blood cell distribution.

**Table 4:** Diagnostic performance of URD ≥ 25% for detection of glomerular hematuria across different biopsy-proven glomerular disease subtypes.

Glomerular Diseases	*n*	Sensitivity (%)	Specificity (%)
IgA nephropathy	20	85	89
Lupus nephritis	32	93.8	89
Pauci-immune GN	13	92.3	89
FSGS	9	88.9	89
Membranous nephropathy	8	100.0	89
Others	19	89.5	89

Abbreviations: GN, glomerulonephritis; FSGS, focal and segmental glomerulosclerosis.

**Table 5:** Comparison of diagnostic performance between URD and conventional urine microscopy.

(**A**) Diagnostic performance.					
**Method**	**Sensitivity**	**Specificity**	**AUC**	**95% CI**	***p* Value**
URD ≥ 25%	91.1	89.0	0.905	0.850–0.938	<0.0001
Technician	60.4	81.0	0.707	0.639–0.769	<0.0001
Nephrology fellow	83.2	90.0	0.886	0.811–0.910	<0.0001
(**B**) Pairwise comparison of ROC curves					
**Comparison**		**Difference in AUC (95% CI)**			***p* Value**
URD ≥ 25% vs. technician		0.194 (0.124–0.263)			<0.0001
URD ≥ 25% vs. fellow		0.035 (−0.023–0.092)			0.2374
Technician vs. fellow		0.159 (0.105–0.212)			<0.0001
(**C**) Agreement between conventional urine microscopy, URD ≥ 25%, and biopsy-confirmed diagnosis assessed by Cohen’s kappa coefficient					
**Comparison**		**Cohen’s κ (95% CI)**			
Technician vs. fellow		0.637 (0.532–0.741)			
Technician vs. biopsy diagnosis		0.414 (0.287–0.533)			
Fellow vs. biopsy diagnosis		0.731 (0.631–0.821)			
URD ≥ 25% vs. biopsy diagnosis		0.801 (0.720–0.871)			
Technician vs. URD ≥ 25%		0.396 (0.254–0.509)			
Fellow vs. URD ≥ 25%		0.652 (0.544–0.752)			

For URD ≥ 25%, AUC represents the diagnostic performance of the dichotomized cutoff. The continuous URD parameter demonstrated an AUC of 0.946 in ROC analysis. Abbreviations: URD, urinary red blood cell distribution; AUC, area under the curve; ROC, receiver operating characteristic.

## Data Availability

The original contributions presented in this study are included in the Supplementary Materials. Further inquiries can be directed to the corresponding author.

## Supplementary Materials

The following supporting information can be downloaded at https://www.mdpi.com/article/10.3390/diagnostics16162491/s1, Table S1: Internal validation of the study-derived URD ≥ 25% cutoff using bootstrap resampling; Table S2: Associations between UF-5000 urinary red blood cell parameters and glomerular hematuria; Table S3: Variance inflation factor analysis for the multivariable logistic regression model; Table S4: Multivariable logistic regression analysis using the study-derived URD cutoff of ≥25%.; Table S5: Sensitivity analysis of the multivariable logistic regression model including blood urea nitrogen; Table S6: Diagnostic performance of URD ≥ 25% for detecting glomerular hematuria across urinary RBC count strata; Table S7: Distribution of acanthocyte grades assessed by technician- and fellow-performed urine microscopy according to study group.
