# Supplementary material for: Urinary Red Blood Cell Distribution for Detection of Glomerular Hematuria: A Biopsy-Based Comparison with Direct Microscopy
Source: Diagnostics (Basel). 2026 Aug 7;16(16):2491. doi: 10.3390/diagnostics16162491 (PMC13511529; doi:10.3390/diagnostics16162491)
Supplement: Supplementary file 1 [file diagnostics-16-02491-s001.zip › diagnostics-4430612-supplementary.pdf]

# Supplement Material

**Urinary Red Blood Cell Distribution for Detection of Glomerular Hematuria: A Biopsy-Based Comparison with Direct Microscopy**

Nuttasith Larpparisuth, Paweena Kanogpotjananont, Kanit Reesukumal, Methawoot Khemmongkol, Gahwin Ruchikajorndech, Chanchira Janwijit, Peenida Skulratanasak,

**Supplementary Table S1:** Internal validation of the study-derived URD ≥25% cutoff using bootstrap resampling

| **Diagnostic parameter** | **Estimate** | **Bootstrap 95% CI** |
|---|---|---|
| AUC | 0.946 | 0.910 – 0.975 |
| Sensitivity (%) | 91.1 | 85.0 – 96.2 |
| Specificity (%) | 89.0 | 82.3 – 94.7 |

Internal validation was performed using 5,000 bootstrap resamples.

**Abbreviations**: AUC, area under the curve

**Supplementary Table S2:** Associations between UF-5000 urinary red blood cell parameters and glomerular hematuria

| Para-meters | Ideal cut off value | Sensi tivity (%) | Speci ficity (%) | Univariate analysis | | | Multivariate analysis | | |
|---|---|---|---|---|---|---|---|---|---|
| | | | | Exp (B) | 95% CI | P value | Exp (B) | 95% CI | P value |
| URD (%) | 25 | 91.1 | 89 | 4.94 | 0.55 – 44.8 | 0.156 | 9.09 | 2.28 – 36.19 | 0.002 |
| Small RBC (%) | 35 | 87.1 | 88 | 0.92 | 0.12 – 6.99 | 0.939 | | | |
| Lysed RBC (%) | 2 | 84.2 | 78 | 4.79 | 1.52 – 15.07 | 0.007 | 5.13 | 1.69 – 15.59 | 0.004 |
| RBC-P70 Fsc (ch) | 125 | 81.9 | 94.8 | 12.87 | 2.76 – 59.9 | 0.001 | 11.3 | 2.6 – 49.36 | 0.001 |
| RBC-SF-Fsc-DW (ch) | 40 | 79.5 | 80.4 | 2.77 | 0.76 – 10.03 | 0.121 | | | |

**Abbreviations**: URD, urinary red blood cell distribution; Small RBC (%), percentage of small red blood cells; Lysed RBC (%), percentage of lysed red blood cells; RBC-P70 Fsc, RBC-P70 forward-scattered light; RBC-SF-Fsc-DW, RBC surface-channel forward-scattered light distribution width

**Supplementary Table S3:** Variance inflation factor analysis for the multivariable logistic regression model

| **Variable** | **VIF** |
| --- | --- |
| URD | 2.41 |
| Age | 2.93 |
| Female sex | 2.27 |
| Diabetes mellitus | 1.40 |
| Malignancy | 1.24 |
| Serum creatinine | 2.01 |
| Urine protein | 1.95 |

**Abbreviations**: URD, urinary red blood cell distribution

**Supplementary Table S4:** Multivariable logistic regression analysis using the study-derived URD cutoff of ≥25%.

| Variable | Adjusted OR | 95% CI | P |
|---|---|---|---|
| URD ≥25% | 58.4 | 18.7–182.5 | <0.001 |
| Age | 0.98 | 0.94–1.02 | 0.291 |
| Female sex | 0.81 | 0.18–3.64 | 0.784 |
| Diabetes mellitus | 0.35 | 0.07–1.78 | 0.207 |
| Malignancy | 0.09 | 0.01–0.96 | 0.046 |
| Serum creatinine | 1.10 | 0.85–1.42 | 0.474 |
| UPCR | 2.18 | 1.33–3.57 | 0.002 |

All variance inflation factors were <5.0, indicating no significant multicollinearity.

**Abbreviations**: URD, urinary red blood cell distribution; UPCR, urine protein creatinine ratio

**Supplementary Table S5:** Sensitivity analysis of the multivariable logistic regression model including blood urea nitrogen.

| **Variable** | **Adjusted OR** | **95% CI** | **P** |
|---|---|---|---|
| URD | 1.07 | 1.04–1.09 | <0.001 |
| Age | 0.98 | 0.94–1.02 | 0.291 |
| Female sex | 0.74 | 0.17–3.22 | 0.689 |
| Diabetes mellitus | 0.26 | 0.05–1.31 | 0.102 |
| Malignancy | 0.11 | 0.01–1.11 | 0.061 |
| Serum creatinine | 1.13 | 0.78–1.65 | 0.515 |
| UPCR | 1.82 | 1.21–2.75 | 0.004 |
| BUN | 1.00 | 0.97–1.03 | 0.888 |

All variance inflation factors were <5.0, indicating no significant multicollinearity.

**Abbreviations**: URD, urinary red blood cell distribution; UPCR, urine protein creatinine ratio; BUN, blood urea nitrogen

**Supplementary Table S6**: Diagnostic performance of URD ≥25% for detecting glomerular hematuria across urinary RBC count strata

| RBC count (cells/µL) | N | GH group (N) | NGH group (N) | AUC (95% CI) | Sensitivity (%) | Specificity (%) |
|---|---|---|---|---|---|---|
| <50 | 20 | 18 | 2 | 1.00 (0.83 – 1.00) | 94.4 | 100.0 |
| 50–200 | 49 | 35 | 14 | 0.96 (0.89 – 1.00) | 94.3 | 92.9 |
| >200 | 132 | 48 | 84 | 0.93 (0.89 – 0.98) | 87.5 | 88.1 |

**Supplementary Table S7**: Distribution of acanthocyte grades assessed by technician- and fellow-performed urine microscopy according to study group

| Acanthocyte grade | Technician | | Fellow | |
|---|---|---|---|---|
| | GH group (N, %) | NGH group (N, %) | GH group (N, %) | NGH group (N, %) |
| 0% | 28 (27.7) | 52 (52.0) | 8 (7.9) | 51 (51.0) |
| <5% | 12 (11.9) | 29 (29.0) | 9 (8.9) | 39 (39.0) |
| 5–10% | 11 (10.9) | 6 (6.0) | 21 (20.8) | 6 (6.0) |
| 10–20% | 13 (12.9) | 4 (4.0) | 33 (32.7) | 3 (3.0) |
| 20–40% | 17 (16.8) | 3 (3.0) | 19 (18.8) | 0 (0.0) |
| >40% | 20 (19.8) | 6 (6.0) | 11 (10.9) | 1 (1.0) |
